# Effectiveness of Single-Component Clinician Reminder Systems for Smoking and Obesity Preventive Care in Primary Care: A Systematic Review

**DOI:** 10.7759/cureus.108442

**Published:** 2026-05-07

**Authors:** Mohammed A Khormi, Mubarak A Alqahtani, Halimah M Mujayri, Ali O Alzahrani, Ghada A Alqarni, Renad A Alshehri, Sarah S Alaklabi, Alhanouf W Alharthi, Alanoud O Alharthi, Arwa O Alharthi, Asma F Alshahrani, Mohammed K Zughlul

**Affiliations:** 1 Department of Family Medicine, Jazan Health Cluster, Jazan, SAU; 2 College of Medicine, University of Bisha, Bisha, SAU; 3 College of Medicine, Jazan University, Jazan, SAU; 4 College of Medicine, University of Jeddah, Jeddah, SAU

**Keywords:** behavioral risk factors, clinical decision support systems, clinician reminder systems, electronic medical records, obesity management, preventive care, primary care, smoking cessation, systematic review

## Abstract

Clinician-facing reminder systems are widely used in primary care to improve preventive care delivery, but their effectiveness in managing behavioral risk factors remains uncertain. This systematic review and meta-analysis evaluated whether single-component clinician reminder systems improve smoking- and obesity-related preventive care in primary care settings. Four electronic databases were searched from inception to May 2026 for primary care studies comparing single-component clinician reminder systems with usual care. Eligible study designs included randomized controlled trials, cluster randomized controlled trials, and controlled before-and-after studies. Risk of bias was assessed using the Cochrane Risk of Bias 2 (RoB 2) tool and the Risk Of Bias In Non-randomized Studies of Interventions (ROBINS-I) tool. A random-effects meta-analysis was conducted using risk ratios with 95% confidence intervals (CIs). Four studies conducted in the United States between 1995 and 2022 were included; three examined smoking-related reminders, and one evaluated an obesity documentation prompt. Two studies were judged to have low risk of bias, one had some concerns, and one had a serious risk of bias. Individual studies generally demonstrated improvements in at least one preventive care process outcome: smoking status vital sign prompts increased physician discussion of smoking from 47% to 86% and advice to quit from 50% to 80%; routine smoking identification increased smoking cessation counseling from 53.4% to 61.9% and advice to quit from 51.5% to 59.9%; an electronic best practice alert increased quitline referrals from 0% in usual care to 9.1% in the intervention group; and adding obesity to the problem list increased documentation of obesity being addressed during follow-up from 4.6% to 14.7%. Despite these favorable individual findings, pooled analysis showed no statistically significant overall effect, with very high heterogeneity across studies. In conclusion, single-component clinician reminder systems may improve selected preventive care processes in primary care, but the overall evidence remains heterogeneous and inconclusive.

## Introduction and background

Smoking and obesity are among the leading preventable causes of morbidity and premature mortality and are highly relevant to primary care practice [1,2]. Tobacco use causes more than seven million deaths annually worldwide, while obesity affects over one billion people globally and continues to increase [1-4]. Primary care provides repeated opportunities for prevention through routine identification of smoking and excess weight, enabling counseling, referral, and follow-up [3,5]. Although interventions targeting smoking cessation, obesity, alcohol use, and physical inactivity in primary care are effective and cost-effective, their implementation in routine practice remains suboptimal [1-5].

The gap between evidence and delivery is particularly evident in smoking cessation counseling and obesity management. Evidence shows that delivery of the smoking cessation “5As” framework (ask, advise, assess, assist, and arrange) declines at more intensive stages, with assist and arrange performed less consistently than ask and advise [1-6]. Barriers such as limited consultation time, high workload, and insufficient clinician training continue to limit preventive counseling in routine care. Addressing all recommended preventive services would require substantial additional clinician time, further constraining implementation [6-10].

Clinician-facing reminder systems have been proposed as a practical strategy to improve preventive care delivery. These systems, which may be electronic or paper-based, prompt actions such as documenting smoking status, providing cessation counseling, recording body mass index (BMI), identifying obesity, or making referrals at the point of care. Prior reviews have reported generally positive but variable effects of reminders on preventive care processes, with effectiveness influenced by workflow integration, usability, and whether reminders are used alone or as part of multicomponent interventions [7-11].

However, existing evidence is fragmented, and many reviews combine reminder systems with other implementation strategies or multiple risk behaviors, making it difficult to isolate the effect of single-component clinician reminders. This systematic review and meta-analysis therefore assessed whether single-component clinician-facing reminder systems improve smoking- and obesity-related preventive care in adult primary care settings.

## Review

Methods

This systematic review and meta-analysis was conducted and reported in accordance with the Preferred Reporting Items for Systematic Reviews and Meta-Analyses (PRISMA) guidelines [12].

Search Strategy and Study Selection

A comprehensive search was conducted in four databases (MEDLINE, Scopus, Web of Science, and the Cochrane Library) from inception to March 1, 2026. The search strategy combined terms related to reminder systems and primary care. All retrieved records were imported into EndNote Reference Manager (Clarivate, London, UK) for duplicate removal. Titles, abstracts, and full texts were screened independently by two reviewers, with disagreements resolved by a third senior reviewer. Reference lists of included studies were also screened to identify additional eligible studies (Tables 1, 2).

**Table 1:** PICO framework for the systematic review This table outlines the Population, Intervention, Comparator, and Outcomes (PICO) framework used to define eligibility criteria for the systematic review. The population includes adults attending primary care settings. The intervention consists of single-component clinician-facing reminder systems aimed at improving preventive care delivery. Comparators include usual care or no reminder systems. Outcomes focus on preventive care processes related to smoking and obesity, including screening, counseling, documentation, and referral activities. Eligible study designs included randomized controlled trials (RCTs), cluster RCTs, and controlled before-and-after studies. EMR: electronic medical record; BMI: body mass index

Component	Description
Population (P)	Adults (≥18 years) attending primary care settings (e.g., general practice, family medicine, and community primary care)
Intervention (I)	Single-component clinician-facing reminder systems (e.g., electronic alerts, paper prompts, vital sign stamps, and EMR prompts) targeting smoking status or obesity-related preventive care
Comparator (C)	Usual care or no reminder system
Outcomes (O)	Preventive care process outcomes related to smoking (e.g., identification, counseling, and quitline referral) and obesity (e.g., BMI recording, documentation, counseling, and follow-up care)
Study design	RCTs, cluster RCTs, and controlled before-and-after studies

**Table 2:** Search strategy used for electronic database searches This table summarizes the key search terms and concepts used to construct the electronic database search strategy. The search combined terms related to primary care settings, clinician reminder systems, smoking-related outcomes, and obesity-related outcomes. Boolean operators (AND/OR) were used to combine concepts. Synonyms and variations were included to maximize sensitivity. The strategy was adapted as appropriate for each database (MEDLINE, Scopus, Web of Science, and Cochrane Library) and applied from database inception to March 1, 2026.

Concept	Keywords/search terms
Population/setting	“primary care” OR “family practice” OR “general practice” OR “ambulatory care” OR “community health services”
Intervention	“clinician reminder” OR “clinical reminder system” OR “decision support system” OR “electronic reminder” OR “prompt*” OR “alert*” OR “best practice alert” OR “vital sign reminder”
Smoking outcomes	“smoking” OR “tobacco use” OR “smoking cessation” OR “quit smoking” OR “tobacco counseling”
Obesity outcomes	“obesity” OR “overweight” OR “body mass index” OR “BMI” OR “weight management”
Study design filter (if used)	“randomized controlled trial” OR “cluster randomized” OR “controlled before and after”

Eligibility Criteria

We included studies involving adults (≥18 years) attending primary care settings, defined as the first point of contact within the healthcare system, including general practice, family medicine, and community-based primary care services. Studies including adolescents were eligible only if data for adults could be extracted separately.

Eligible interventions were single-component clinician-facing reminder systems delivered at the point of care, including electronic alerts, paper-based prompts, vital sign prompts, and problem-list reminders. Eligible outcomes were limited to preventive care processes targeting behavioral risk factors, specifically smoking (e.g., smoking status assessment and cessation counseling) and obesity (e.g., BMI recording, obesity documentation, and weight management counseling).

Comparators included usual care or no reminder intervention. Eligible study designs were randomized controlled trials (RCTs), cluster RCTs, and controlled before-and-after studies. We excluded studies involving inpatient, specialty, or palliative care populations, as well as studies evaluating multicomponent interventions combining reminders with additional strategies such as education, workflow redesign, or organizational change. No restrictions were applied for language or publication date.

Data Extraction and Quality Assessment

Two reviewers independently extracted data using a standardized Excel spreadsheet (Microsoft Corp., Redmond, WA, USA). Extracted variables included study characteristics (design, setting, sample size, and inclusion/exclusion criteria), participant characteristics, intervention and comparator details, and preventive care outcomes.

Risk of bias was assessed using the Cochrane Risk of Bias 2 (RoB 2) tool [13], including its cluster RCT extension, for randomized studies, and the Risk Of Bias In Non-randomized Studies of Interventions (ROBINS-I) tool for non-randomized studies [14]. RoB 2 evaluates bias across randomization, deviations from intended interventions, missing data, outcome measurement, and selective reporting. ROBINS-I assesses bias due to confounding, participant selection, intervention classification, deviations from intended interventions, missing data, outcome measurement, and selective reporting.

Statistical Analysis

All analyses were conducted using R software version 4.4 (R Foundation for Statistical Computing, Vienna, Austria). Dichotomous outcomes were extracted as event counts and total participants per group. Pooled effects were estimated using risk ratios (RRs) with 95% confidence intervals (CIs) under a random-effects model. Statistical significance was defined as a two-sided p-value < 0.05. Heterogeneity was assessed using Cochran’s Q test and the I² statistic, with p < 0.10 and I² ≥ 50% indicating substantial heterogeneity. Sensitivity analyses included leave-one-out analyses and Baujat plots to assess the influence of individual studies. Small-study effects and publication bias were evaluated using Doi plots and the Luis Furuya-Kanamori (LFK) index, where values within ±1 indicate no asymmetry, ±1 to ±2 indicate minor asymmetry, and beyond ±2 indicate major asymmetry. Due to variability in reported outcomes, all eligible endpoints were grouped as preventive care process measures and pooled under the assumption that they reflect clinician adherence to recommended preventive practices.

Results

Literature Search and Study Selection

**Figure 1:**
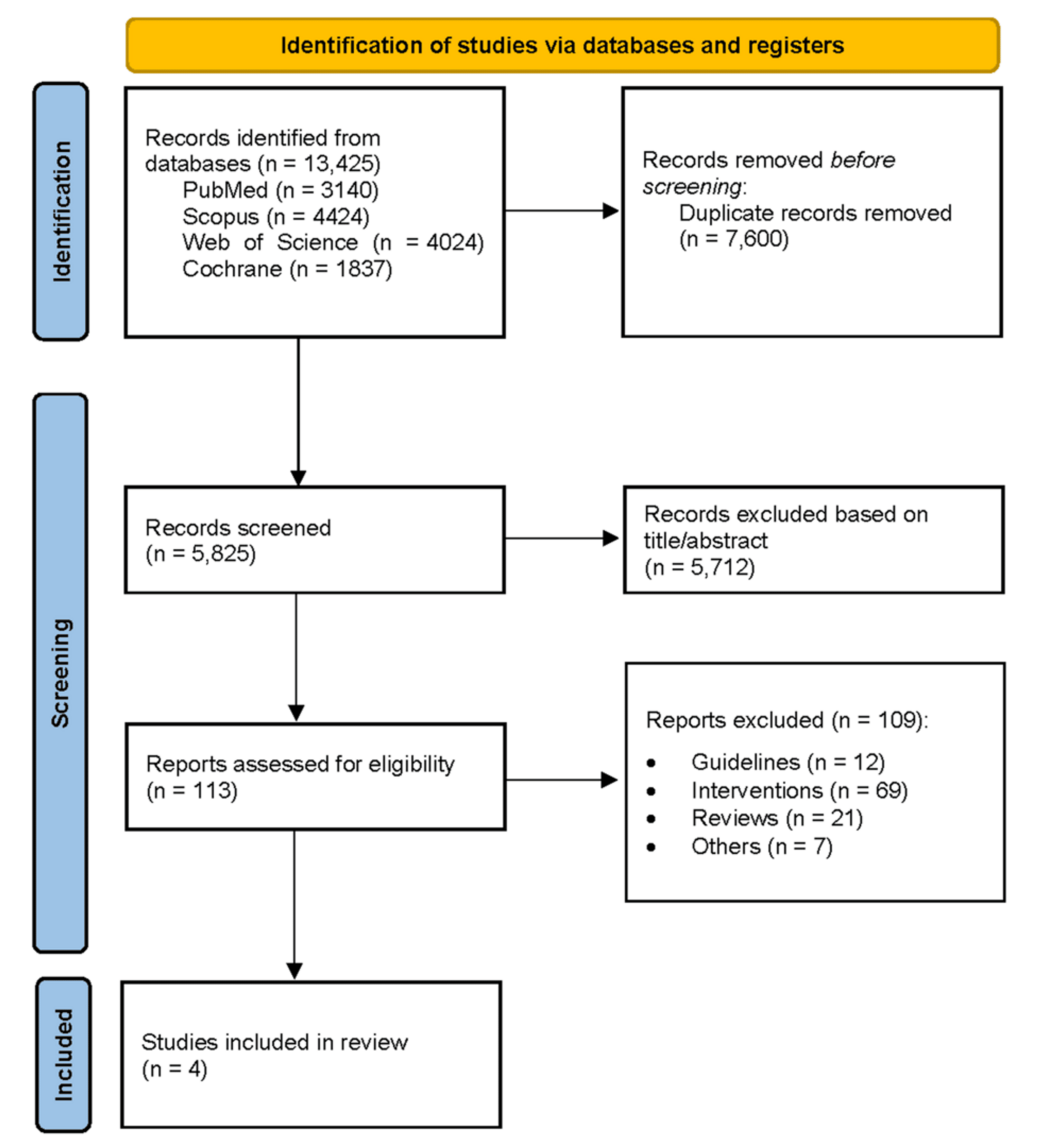
PRISMA flow diagram depicting the study selection process for the systematic review PRISMA (Preferred Reporting Items for Systematic Reviews and Meta-Analyses) flow diagram detailing the study selection process for this systematic review [12]. Following the identification of records through database searching and other sources, duplicates were removed, and the remaining records were screened. Full-text articles were assessed for eligibility, with exclusions documented along with reasons. Studies meeting the inclusion criteria were included in the final synthesis.

Database searches identified 13,425 records. After removing duplicates using EndNote, 5,825 records remained for screening. Following title, abstract, and full-text screening, four studies were included in the systematic review and meta-analysis (Figure 1) [1-4].

Characteristics of the Included Studies

Four studies published between 1995 and 2022 were included, all conducted in the United States. Study designs included one before-and-after study, one RCT, and two cluster RCTs. Three studies evaluated smoking-related clinician prompts, while one evaluated an obesity-related documentation prompt in primary care (Tables 3, 4).

**Table 3:** Summary of included studies evaluating clinician-facing reminder systems for smoking and obesity preventive care in primary care This table summarizes the characteristics and key findings of studies included in the systematic review evaluating single-component clinician-facing reminder systems in primary care for smoking cessation and obesity-related preventive care. The table includes study design, patient population, eligibility criteria, exclusion criteria, sample size, details of the intervention and comparator, and the main study conclusion. BMI: body mass index; BPA: best practice alert; EMR: electronic medical record

Study ID	Study design	Population	Inclusion criteria	Exclusion criteria	Total sample size	Intervention	Control	Conclusion
Rothemich et al. [1]	Cluster randomized controlled trial	Adult patients exiting primary care practices	Age ≥ 18 years and seen by a clinician during study visits	Patients refusing, too ill, or with language/vision difficulties; non-responders; practices in residencies or special populations; practices already using systematic tobacco identification	6,729	Rooming staff used a vital sign rubber stamp to ask all adult patients about tobacco use and record responses within the stamp	Usual routine care without systematic tobacco identification or reminder system	The intervention produced a modest increase in simple advice to quit but did not increase intensive smoking cessation counseling
Banerjee et al. [2]	Randomized controlled trial	Obese adult patients in a family medicine residency clinic	Patients aged 18-64 years with a visit in the previous year, BMI ≥ 30, at least one appointment during 5-month follow-up, and no obesity listed on the problem list	Pregnant patients; patients with obesity addressed in the past year; no follow-up visits; providers aware of the study	497	Manual addition of obesity to the patient’s problem list by the research team	Usual medical record without obesity diagnosis added	Adding obesity to the problem list significantly increased the likelihood of providers addressing obesity at subsequent visits
Wadlin et al. [3]	Cluster randomized controlled trial	Adult patients identified as current smokers in primary care clinics	Primary care encounters for adults ≥ 18 years labeled in the EMR as current smokers	Encounters where the BPA was triggered for other reasons (e.g., nursing encounters or medication refills)	19,930	EMR-based BPA prompting electronic referral to a state quitline (without provider education)	Waitlist control receiving usual care	The BPA increased quitline referrals, although further work is needed to improve provider utilization of quitlines and patient acceptance
Robinson et al. [4]	Before-and-after study	Adult ambulatory patients in a metropolitan family practice residency program	Adult patients ≥ 18 years presenting to the billing clerk at exit from the family practice center	Patients completing more than one survey (only first analyzed); no attempt to contact non-responders	637	Introduction of a vital sign stamp recording smoking status in the chart and prompting nurses to ask about smoking at each visit	Usual care prior to implementation of the stamp	Including smoking as a vital sign significantly increased the likelihood of smoking-related discussions between patients and physicians

**Table 4:** Baseline characteristics of included studies This table presents the baseline characteristics of participants included in studies evaluating single-component clinician-facing reminder systems for smoking cessation and obesity-related preventive care in primary care settings. Data are stratified by intervention and control groups within each study and include sample size, mean age, sex distribution, unit of analysis, clinical focus of the intervention, and relevant baseline clinical characteristics (e.g., smoking status or obesity status where applicable). Where reported, standard deviations (SDs) are provided for continuous variables. BMI: body mass index; EMR: electronic medical record; n: number of participants; NA: not available

Study ID	Groups	Sample size n (%)	Age, mean (SD)	Female, n (%)	Unit analyzed	Clinical focus	Key baseline status
Rothemich et al. [1]	Intervention	2,881 (42.8)	50.6 (11.87)	550 (60.0)	Patients	Smoking status as a vital sign	Current smokers: 180 (19.6)
	Control	3,848 (57.2)	53 (11.57)	740 (60.3)			Current smokers: 205 (16.7)
Banerjee et al. [2]	Intervention	258 (51.9)	48.0 (16.9)	187 (72.5)	Patient records	Addressing obesity	Obese (BMI ≥ 30): 258 (100); mean BMI: 34.9 (4.8)
	Control	239 (48.1)	46.0 (16.4)	176 (73.6)			Obese (BMI ≥ 30): 239 (100); mean BMI: 34.3 (4.4)
Wadlin et al. [3]	Intervention	6,857 (48.0)	NA	NA	Clinic encounters	EMR-based quitline referral	Current smokers: 6,857 (100)
	Control	7,437 (52.0)	NA	NA			Current smokers: 7,437 (100)
Robinson et al. [4]	Intervention	239 (37.5)	44.3 (16.2)	177 (74)	Patients	Smoking cessation counseling	Current smokers: 84 (35)
	Control	398 (62.5)	44.5 (16.8)	314 (79)			Current smokers: 95 (24)

Robinson et al. [4] evaluated a smoking status vital sign stamp in a family practice residency program (n = 637 patients, including 179 smokers). Rothemich et al. [1] assessed routine smoking status documentation as a vital sign across 18 primary care practices (n = 6,729 patients, including 1,149 smokers). Banerjee et al. [2] examined the addition of obesity to the problem list in a family medicine residency clinic (n = 497 patient records). Wadlin et al. [3] evaluated an electronic medical record (EMR)-based best practice alert (BPA) prompting referral to a state quitline across 22 primary care sites (19,930 BPA-eligible encounters).

Risk-of-Bias Assessment

Using the ROBINS-I tool, Rothemich et al. [1] and Wadlin et al. [3] were judged at low risk of bias, Banerjee et al. [2] had some concerns, and Robinson et al. had a serious risk of bias (Table 5).

**Table 5:** Risk-of-bias assessment of included studies using RoB 2 and ROBINS-I tools This table presents the risk-of-bias assessment of the included studies in the systematic review using the Cochrane RoB 2 tool for randomized and cluster-randomized trials and the ROBINS-I tool for non-randomized studies [13,14]. Each study is assessed across predefined domains (D1-D7), with domain-level and overall judgments reported. RoB 2 evaluates bias arising from the randomization process, deviations from intended interventions, missing outcome data, outcome measurement, and selective reporting. ROBINS-I evaluates bias due to confounding, participant selection, intervention classification, deviations from intended interventions, missing data, outcome measurement, and selective reporting. Overall risk-of-bias judgments are summarized for each study based on domain-level assessments. RoB 2: Risk of Bias 2; ROBINS-I: Risk Of Bias In Non-randomized Studies of Interventions; D1-D7: risk-of-bias domains as specified in the RoB 2 and ROBINS-I tools; N/A: not applicable

Study	Tool	D1	D1b	D2	D3	D4	D5	D6	D7	Overall
Rothemich et al. [1]	RoB 2	Low	Low	Low	Low	Low	Low	N/A	N/A	Low
Banerjee et al. [2]	RoB 2	Low	N/A	Some concerns	Some concerns	Low	Low	N/A	N/A	Some concerns
Wadlin et al. [3]	RoB 2	Low	Low	Low	Low	Low	Low	N/A	N/A	Low
Robinson et al. [4]	ROBINS-I	Serious	N/A	Low	Low	Low	Moderate	Moderate	Low	Serious

Individual Study Findings

In Robinson et al. [4], introducing a smoking status vital sign stamp increased physician discussion of smoking (86% vs. 47%, p < 0.001) and advice to quit smoking (80% vs. 50%, p < 0.001). Nurse-led smoking discussions also increased (77% vs. 23%, p < 0.001), and post-intervention exposure was associated with higher odds of physician smoking discussion (odds ratio (OR) 5.3, 95% CI 2.5-11.2).

In Rothemich et al. [1], routine smoking status documentation increased smoking cessation counseling (61.9% vs. 53.4%, p = 0.04), advice to quit smoking (59.9% vs. 51.5%, p = 0.04), and smoking status inquiry (79.5% vs. 49.4%, p < 0.001). In Wadlin et al. [3], the EMR-based BPA increased quitline referrals compared with usual care (9.1% vs. 0%). The adjusted OR for referral was 12.70 (95% CI 5.03-32.03), and 21% of referred patients accepted quitline services.

In Banerjee et al. [2], adding obesity to the problem list improved documentation and follow-up. Obesity was more frequently addressed when listed on the problem list (55.5% vs. 5.1%, p < 0.001), and during follow-up, it was addressed more often in the intervention group than in controls (14.7% vs. 4.6%, p < 0.001).

Primary Outcome

**Figure 2:**
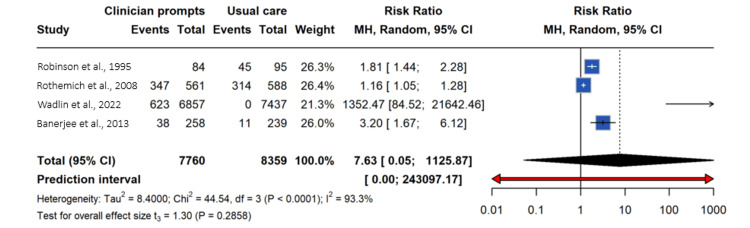
Forest plot of the effect of single-component clinician reminder systems on preventive care outcomes in primary care This forest plot presents the pooled effect of single-component clinician-facing reminder systems compared with usual care across four included studies. Outcomes include smoking-related preventive care processes (smoking counseling, quitline referral, and smoking documentation) and obesity-related follow-up care. Effect sizes are reported as risk ratios (RRs) with 95% confidence intervals (CIs) using a random-effects model. The size of each square represents the weight of the individual study in the meta-analysis, and horizontal lines indicate 95% CIs. The diamond represents the overall pooled effect estimate. Substantial heterogeneity was observed among studies (I² = 93.3%), reflecting variability in intervention types, settings, and outcome definitions.

All four studies were included in the meta-analysis. The pooled analysis using a random-effects model showed no statistically significant difference between clinician reminders and usual care (RR 7.63, 95% CI 0.05-1,125.87, p = 0.29), with high heterogeneity (I² = 93.3%, p = 0.0001). Sensitivity analysis indicated that no single study materially changed the pooled estimate or reduced heterogeneity (Figure 2).

Discussion

This systematic review and meta-analysis evaluated the effectiveness of single-component clinician-facing reminder systems for improving smoking- and obesity-related preventive care in primary care. While individual studies demonstrated improvements in specific process measures-such as smoking counseling, quitline referrals, and obesity documentation-the pooled analysis showed no statistically significant overall effect and substantial heterogeneity. These findings suggest that although reminder systems may influence discrete clinician behaviors, their standalone impact on broader preventive care delivery is inconsistent.

The observed improvements in individual studies are consistent with prior literature indicating that clinician reminders can enhance adherence to guideline-recommended preventive practices, particularly for simple, low-effort actions such as documentation or brief advice [6-9]. For example, incorporating smoking status as a vital sign has been shown to increase rates of screening and brief counseling, which aligns with findings from Rothemich et al. and Robinson et al. in this review [1,4]. Similarly, electronic referral systems, such as the EMR-based BPA evaluated by Wadlin et al., have demonstrated potential to increase referrals to smoking cessation services, although uptake and patient engagement remain variable [3,8].

However, the lack of a significant pooled effect highlights important limitations of single-component reminder systems. One key factor is clinical and methodological heterogeneity across studies. The included interventions ranged from paper-based prompts to electronic alerts embedded in modern EMRs, reflecting substantial variation in usability, integration into clinical workflows, and technological sophistication. Previous research has shown that the effectiveness of clinical decision support systems is highly dependent on factors such as timing, relevance, and ease of use, with poorly integrated systems contributing to alert fatigue and reduced clinician responsiveness [7,9].

Another important consideration is the heterogeneity of outcomes pooled in this analysis. The included studies measured different preventive care process outcomes, including smoking counseling, quitline referral, documentation practices, and obesity follow-up. Although these were grouped under the broader construct of clinician preventive care behavior, they differ in clinical significance and proximity to patient outcomes. For instance, documentation of obesity or smoking status represents an early step in care delivery, whereas counseling and referral are more directly linked to behavior change. This conceptual heterogeneity likely contributed to the high statistical heterogeneity observed (I² = 93.3%) and limits the interpretability of the pooled estimate.

Importantly, the findings align with broader evidence suggesting that single-component interventions are generally less effective than multicomponent strategies in changing clinician behavior and improving patient outcomes. Implementation science literature consistently demonstrates that combining reminders with additional strategies-such as clinician education, audit and feedback, workflow redesign, and patient engagement-produces more substantial and sustained improvements in preventive care delivery [7,9,10]. In this context, reminder systems may function best as enabling tools within a broader system-level intervention rather than as standalone solutions.

The temporal context of the included studies also warrants consideration. Earlier studies relied on paper-based systems or less advanced electronic infrastructure, whereas more recent interventions utilize integrated EMR-based alerts. Advances in health information technology, including adaptive and AI-driven decision support systems, may enhance the effectiveness of reminders by improving personalization, reducing unnecessary alerts, and better aligning with clinical workflows. Emerging evidence suggests that more sophisticated systems may overcome some limitations of traditional reminder approaches, although robust comparative studies are still needed [8,9].

This review has several strengths. By focusing specifically on single-component clinician reminder systems, it isolates the independent effect of reminders, which is often difficult to determine in studies evaluating multicomponent interventions. Additionally, the inclusion of both randomized and non-randomized studies provides a broader perspective on real-world effectiveness.

However, several limitations should be acknowledged. The small number of included studies limits statistical power and the precision of the pooled estimate. The substantial heterogeneity in interventions, outcomes, and study designs further limits comparability. Most studies were conducted in the United States and may not be generalizable to other healthcare systems, particularly those with different levels of digital integration. Furthermore, the reliance on process measures rather than patient-centered outcomes (e.g., smoking cessation rates or weight loss) limits conclusions about the clinical impact of these interventions.

Future research should focus on evaluating integrated and adaptive reminder systems within contemporary electronic health record environments. Studies should prioritize patient-centered outcomes and assess long-term effectiveness. Additionally, hybrid implementation-effectiveness designs could help identify how reminder systems can be optimally combined with other strategies to enhance preventive care delivery in routine practice.

## Conclusions

Clinician reminder systems in primary care showed generally positive effects at the individual study level, particularly for improving documentation, counseling, and referral processes. However, the overall pooled evidence was inconsistent due to limited study numbers and substantial heterogeneity. These findings suggest that reminder systems are most likely to influence short-term clinical processes rather than independently driving broader or sustained behavioral change. Overall, clinician reminders appear better suited as part of a broader implementation strategy rather than as standalone interventions.

## References

[1] Rothemich SF, Woolf SH, Johnson RE, Burgett AE, Flores SK, Marsland DW, Ahluwalia JS (2008). Effect on cessation counseling of documenting smoking status as a routine vital sign: an ACORN study. Ann Fam Med.

[2] Banerjee ES, Gambler A, Fogleman C (2013). Adding obesity to the problem list increases the rate of providers addressing obesity. Fam Med.

[3] Wadlin J, Ford DE, Albert MC, Wang NY, Chander G (2022). Implementing an EMR-based referral for smoking quitline services with additional provider education, a cluster-randomized trial. J Gen Intern Med.

[4] Robinson MD, Laurent SL, Little JM Jr (1995). Including smoking status as a new vital sign: it works!. J Fam Pract.

[5] van Westen-Lagerweij NA, Hipple Walters BJ, Potyka F, Croes EA, Willemsen MC (2023). Proactive referral to behavioral smoking cessation programs by healthcare staff: a systematic review. Nicotine Tob Res.

[6] Krist AH, Davidson KW, Mangione CM (2021). Interventions for tobacco smoking cessation in adults, including pregnant persons: US Preventive Services Task Force recommendation statement. JAMA.

[7] Tildy BE, McNeill A, Perman-Howe PR, Brose LS (2023). Implementation strategies to increase smoking cessation treatment provision in primary care: a systematic review of observational studies. BMC Prim Care.

[8] Zehner ME, Kirsch JA, Adsit RT (2022). Electronic health record closed-loop referral ("eReferral") to a state tobacco quitline: a retrospective case study of primary care implementation challenges and adaptations. Implement Sci Commun.

[9] Bailey SR, Albert EL, Seeholzer EL, Lewis SA, Flocke SA (2023). Sustained effects of a systems-based strategy for tobacco cessation assistance. Am J Prev Med.

[10] Mainous AG 3rd, Xie Z, Dickmann SB, Medley JF, Hong YR (2023). Documentation and treatment of obesity in primary care physician office visits: the role of the patient-physician relationship. J Am Board Fam Med.

[11] van den Hout WJ, van Peet PG, Numans ME, Mook-Kanamori DO (2025). Recording practices of body mass index, overweight and obesity by Dutch general practitioners: an observational study. BMC Prim Care.

[12] Page MJ, McKenzie JE, Bossuyt PM (2021). The PRISMA 2020 statement: an updated guideline for reporting systematic reviews. Rev Esp Cardiol (Engl Ed).

[13] Crocker TF, Lam N, Jordão M (2023). Risk-of-bias assessment using Cochrane's revised tool for randomized trials (RoB 2) was useful but challenging and resource-intensive: observations from a systematic review. J Clin Epidemiol.

[14] Sterne JA, Hernán MA, Reeves BC (2016). ROBINS-I: a tool for assessing risk of bias in non-randomised studies of interventions. BMJ.

